# Resumption of ovulation in anovulatory women with PCOS and obesity is associated with reduction of 11β-hydroxyandrostenedione concentrations

**DOI:** 10.1093/humrep/deae058

**Published:** 2024-03-19

**Authors:** Z Wang, M Van Faassen, H Groen, A E P Cantineau, A Van Oers, A Van der Veen, J M Hawley, B G Keevil, I P Kema, A Hoek

**Affiliations:** Department of Obstetrics and Gynecology, University of Groningen, University Medical Center Groningen, Groningen, The Netherlands; Department of Laboratory Medicine, University of Groningen, University Medical Center Groningen, Groningen, The Netherlands; Department of Epidemiology, University of Groningen, University Medical Center Groningen, Groningen, The Netherlands; Department of Obstetrics and Gynecology, University of Groningen, University Medical Center Groningen, Groningen, The Netherlands; Department of Obstetrics and Gynecology, University of Groningen, University Medical Center Groningen, Groningen, The Netherlands; Department of Laboratory Medicine, University of Groningen, University Medical Center Groningen, Groningen, The Netherlands; Department of Clinical Biochemistry, Wythenshawe Hospital, Manchester NHS Foundation Trust, Manchester, UK; Department of Clinical Biochemistry, Wythenshawe Hospital, Manchester NHS Foundation Trust, Manchester, UK; Department of Laboratory Medicine, University of Groningen, University Medical Center Groningen, Groningen, The Netherlands; Department of Obstetrics and Gynecology, University of Groningen, University Medical Center Groningen, Groningen, The Netherlands

**Keywords:** PCOS, lifestyle intervention, resumption of ovulation, anti-Müllerian hormone, androgens, obesity, 11β-hydroxyandrostenedione

## Abstract

**STUDY QUESTION:**

Is resumption of ovulation after a 6-month lifestyle intervention in women with PCOS and obesity associated with differential changes in endocrine and metabolic parameters (weight, insulin resistance, anti-Müllerian hormone (AMH), and androgens) compared to women with PCOS who remained anovulatory?

**SUMMARY ANSWER:**

Resumption of ovulation after a 6-month lifestyle intervention in women with PCOS and obesity is associated with changes in serum 11β-hydroxyandrostenedione (11OHA4) concentrations.

**WHAT IS KNOWN ALREADY:**

Lifestyle interventions have been shown to reduce clinical and biochemical hyperandrogenism in women with PCOS. Weight loss of 5–10% may reverse anovulatory status, thereby increasing natural conception rates. However, the mechanisms underlying why some women with PCOS remain anovulatory and others resume ovulation after weight loss are unclear. Reproductive characteristics at baseline and a greater degree of change in endocrine and metabolic features with lifestyle intervention may be crucial for ovulatory response.

**STUDY DESIGN, SIZE, DURATION:**

We used data and samples originating from an earlier randomized controlled trial (RCT), which examined the efficacy of a 6-month lifestyle intervention prior to infertility treatment compared to prompt infertility treatment on live birth rate in women with obesity. A total of 577 women with obesity (BMI > 29 kg/m^2^) were randomized between 2009 and 2012. Anovulatory women with PCOS who were allocated to the intervention arm of the original RCT (n = 95) were included in the current analysis.

**PARTICIPANTS/MATERIALS, SETTING, METHODS:**

We defined women as having resumed ovulation (RO+) based on the following criteria: spontaneous pregnancy; or assignment to expectant management; or IUI in natural cycles as the treatment strategy after lifestyle intervention. Steroid hormones were measured using liquid chromatography tandem mass spectrometry. Generalized estimating equations with adjustment for baseline measures and interaction between group and time was used to examine differences in changes of endocrine and metabolic parameters between RO+ (n = 34) and persistently anovulatory women (RO−, n = 61) at 3 and 6 months after intervention.

**MAIN RESULTS AND THE ROLE OF CHANCE:**

At baseline, the mean ± SD age was 27.5 ± 3.6 years in the RO+ group and 27.9 ± 4.1 years in the RO− group (*P* = 0.65), and the mean ± SD weights were 101.2 ± 9.5 kg and 105.0 ± 14.6 kg, respectively (*P* = 0.13). Baseline AMH concentrations showed significant differences between RO+ and RO− women (median and interquartile range [IQR] 4.7 [3.2; 8.3] versus 7.2 [5.3; 10.8] ng/ml, respectively). Baseline androgen concentrations did not differ between the two groups. During and after lifestyle intervention, both groups showed weight loss; changes in 11OHA4 were significantly different between the RO+ and RO groups (*P*-value for interaction = 0.03). There was a similar trend for SHBG (interaction *P*-value = 0.07), and DHEA-S (interaction *P*-value = 0.06), with the most pronounced differences observed in the first 3 months. Other parameters, such as AMH and FAI, decreased over time but with no difference between the groups.

**LIMITATIONS, REASONS FOR CAUTION:**

No high-resolution transvaginal ultrasonography was used to confirm ovulatory status at the end of the lifestyle program. The small sample size may limit the robustness of the results.

**WIDER IMPLICATIONS OF THE FINDINGS:**

Reduction of androgen concentrations during and after lifestyle intervention is associated with recovery of ovulatory cycles. If our results are confirmed in other studies, androgen concentrations could be monitored during lifestyle intervention to provide individualized recommendations on the timing of resumption of ovulation in anovulatory women with PCOS and obesity.

**STUDY FUNDING/COMPETING INTEREST(S):**

The study was supported by a grant from ZonMw, the Dutch Organization for Health Research and Development (50-50110-96-518). The Department of Obstetrics and Gynecology of the UMCG received an unrestricted educational grant from Ferring Pharmaceuticals BV, The Netherlands. A.H. reports consultancy for the development and implementation of a lifestyle App MyFertiCoach developed by Ferring Pharmaceutical Company. All other authors have no conflicts to declare.

**TRIAL REGISTRATION NUMBER:**

The LIFEstyle RCT was registered at the Dutch trial registry (NTR 1530).

## Introduction

Polycystic ovary syndrome (PCOS) is the leading cause of anovulatory infertility, characterized by chronic anovulation, hyperandrogenemia, and polycystic ovaries. The available evidence suggests that more than one pathway is involved in the pathophysiological mechanisms leading to anovulation in PCOS (Franks and Hardy, 2020). Among these pathways, androgens play a key role in linking hypothalamic or pituitary dysfunction to ovarian dysfunction (Franks and Hardy, 2020). The production of excess androgens in PCOS is complex and has a heterogeneous pathophysiological background. Excess androgen levels increase the number of small antral follicles by stimulating the formation of primordial, primary, secondary and early preantral follicles but without progression to the dominant follicle as a result of compromised granulosa cell function (Lebbe and Woodruff, 2013).

**Figure 1.**
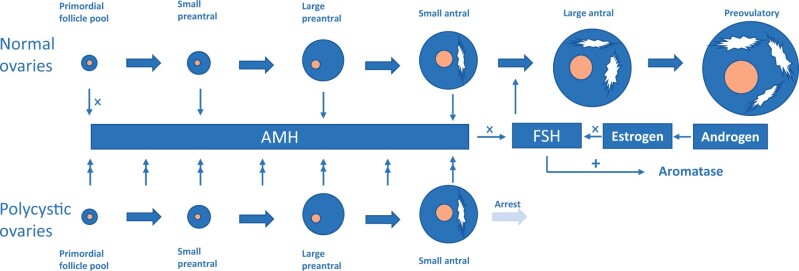
**Schematic of anti-Müllerian hormone actions in normal and polycystic ovaries**. Upper panel: normal ovaries, lower panel: polycystic ovaries. The yellow center represents the oocyte, the blue area represents the granulosa cell layer and the white area represents follicle fluid in the antrum. High levels of anti-Müllerian hormone (AMH) in polycystic ovary syndrome (PCOS) cause increased suppression of FSH signaling and result in arrest in follicular growth.

Anti-Müllerian hormone (AMH), a glycoprotein hormone, is expressed by granulosa cells of growing follicles from the primary up to the small antral stage, and AMH expression in granulosa cells disappears after FSH-dependent selection (Broer *et al.*, 2014). Serum AMH has been recognized as a marker of ovarian function, particularly in assessing ovarian reserve (Moolhuijsen and Visser, 2020). It has been suggested that elevated AMH concentrations may result in acceleration of maturation and premature luteinization of antral follicles, which contributes to problems with ovulation and fertility in PCOS (Dumont *et al.*, 2015). Fig. 1 shows AMH actions in normal and polycystic ovaries.

**Figure 2.**
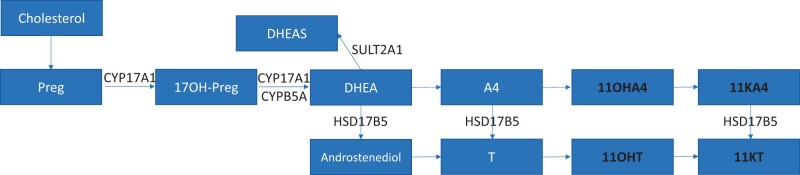
**Schematic of 11-oxygenated androgen synthesis in adipose tissue**. 11-oxygenated androgen synthesis is indicated by bold text. Preg: pregnenolone; 17OH-Preg: 17-hydroxypregnenolone; DHEA(S): dehydroepinadrosterone (sulfate); A4: androstenedione; T: testosterone; 11OHA4,:11β-hydroxyandrostenedione, 11KA4: 11-ketoandrostenedione; 11OHT: 11β-hydroxytestosterone; 11KT: 11-ketotestosterone; CYP17A1: 17α-hydroxylase/17,20-lyase; CYB5A: cytochrome b5 type A; SULT2A1: sulfotransferase 2A1; HSD17B5: 17β-hydroxysteroid dehydrogenase type 5.

Up to 60% of women with PCOS are overweight or obese (Lim *et al.*, 2012). Excess body weight and visceral fat promote the development of insulin resistance and compensatory hyperinsulinemia, which stimulates the synthesis of androgens in the ovaries (Diamanti-Kandarakis and Dunaif, 2012). Elevated androgen concentrations in turn disrupt glucose and insulin regulation (Baptiste *et al.*, 2010). In addition, the production of sex hormone-binding globulin (SHBG) is reduced, which results in an increase in ‘bioavailable’ androgens (Zhu *et al.*, 2019). Women with PCOS and obesity are at high risk of chronic diseases, such as metabolic syndrome and cardiovascular diseases (Anagnostis *et al.*, 2018). Moreover, adipose tissue is an important site for steroid storage and metabolism (Li *et al.*, 2015). An important link between androgen excess and obesity in fat cells is the conversion of androstenedione (A4) to testosterone (T) and 11-ketoandrostenedione (11KA4) to 11-ketotestosterone (11KT) through the activity of 17β-hydroxysteroid dehydrogenase type 5 (HSD17B5) (Fig. 2) (Torchen *et al.*, 2020).

Recent international evidence-based guidelines on PCOS highlight the important role of diet and exercise in the management of women with PCOS, particularly in those who are overweight or obese (Teede *et al.*, 2018). Lifestyle interventions have been shown to reduce clinical and biochemical hyperandrogenism in women with PCOS (Lim *et al.*, 2019). Even a 5–10% loss of weight may reverse anovulatory status, thereby increasing natural conception rates (van Oers *et al.*, 2016). However, the underlying mechanisms of why some women with PCOS remain anovulatory and others resume ovulation after weight loss are unclear. Reproductive characteristics at baseline and a greater degree of change in endocrine and metabolic features with lifestyle intervention in some women may be crucial for ovulatory response (Jarrett and Lujan, 2016). To illustrate, women with lower AMH and higher SHBG at baseline more often experience sporadic ovulations compared to women who remained anovulatory after weight loss, as shown in previous small studies (van Dam *et al.*, 2004; Moran *et al.*, 2007; Thomson *et al.*, 2009). Furthermore, a reduction in BMI, hyperandrogenism and insulin resistance might mediate the occurrence of ovulation resumption in women with PCOS and obesity undergoing weight loss (Pasquali *et al.*, 2006).

In a previously published pilot study of our lifestyle intervention (Kuchenbecker *et al.*, 2011), we investigated body fat distribution, particularly intra-abdominal and abdominal subcutaneous fat, in a group of anovulatory women with PCOS and obesity. We found that women who regained ovulation lost more weight and intra-abdominal fat than those who remained anovulatory after lifestyle intervention (Kuchenbecker *et al.*, 2011). In the current *post hoc* study, we aimed to investigate if resumption of ovulation after a 6-month lifestyle intervention in women with PCOS and obesity was associated with changes in endocrine and metabolic parameters (weight, insulin resistance, AMH, and androgens). It is hypothesized that achieving a certain level of weight loss can lead to a clinically relevant decrease in AMH levels and an improvement in hyperandrogenism, which are essential for resumption of ovulation.

## Materials and methods

### Study design and population

We used samples originating from a multi-center randomized controlled trial (RCT). The RCT was approved by the medical ethics committee of the University Medical Center Groningen (METc code: 2008/284), in combination with approval of the board of directors from the other 22 participating hospitals. All women included in the study provided written informed consent. The study was registered in the Netherlands Trial Registry (NTR 1530). Briefly, a total of 577 infertile women with a BMI > 29 kg/m^2^ were randomly assigned to either a 6-month lifestyle intervention followed by 18 months of fertility treatment or 24 months of fertility treatment between 2009 and 2012, to investigate the effect of lifestyle intervention preceding fertility treatment on live birth (Mutsaerts *et al.*, 2010, 2016). The lifestyle intervention consisted of an energy-restricted diet (a reduction of 600 kcal/day but at least 1200 kcal/day), an increase in physical activity (10 000 steps daily and two to three moderate-to-vigorous sessions of exercise a week) and motivational counseling, with a goal of weight reduction of at least 5% of the original body weight or a reduction in BMI to below 29 kg/m^2^ within the intervention period. All women underwent fertility investigation before randomization and were categorized as unexplained infertility, tubal factor, male factor, anovulation (World Health Organization (WHO) I; WHO II: PCOS; WHO II: non-PCOS), endometriosis, and cervix factor. Women were diagnosed as PCOS if the Rotterdam criteria were met: oligo-ovulation or anovulation, clinical manifestations of hyperandrogenism and/or hyperandrogenemia, or ovarian polycystic changes (Rotterdam ESHRE/ASRM-Sponsored PCOS consensus workshop group, 2004).

Anovulatory women with PCOS who were allocated to the intervention arm of the original RCT were included in the current analysis. They were divided into two groups based on their ovulation status: resumed ovulation (RO+) or remaining anovulatory (RO−) at the end of intervention (6 months after randomization). Women who conceived did not continue in the lifestyle program. Women who stopped the lifestyle program before the sixth month were considered as dropouts. Numbers of available serum hormone measurements decreased over the course of the study period owing to the nature of the RCT with dropouts, pregnancies, failure to visit the hospital, or blood samples being exhausted for other measurements.

### Resumption of ovulation

After completion of the intervention (6 months or in case their weight reduction goal of decrease in body weight was met), women were evaluated to determine any further infertility treatment strategy. During the lifestyle intervention women would monitor their menstrual cycle pattern. Based on information available at the end of the intervention period, we defined women as having resumed ovulation using the following criteria: if they became pregnant spontaneously before the end of the intervention period; if the infertility treatment strategy was expectant management, which is common practice in the Netherlands in ovulatory women with good partner semen quality and no tubal infertility; or, if IUI treatment in natural cycles was started. Women who did not fulfill these criteria were classified as ‘remaining anovulatory’ after the lifestyle intervention.

### Clinical and laboratory measurements

During the hospital visits at randomization, and at 3 and 6 months after randomization, body weight in kg, height in cm, and waist and hip circumference in cm were measured by research nurses not involved in the lifestyle intervention coaching. Fasting blood samples were collected by venipuncture into a collection tube. Cardiometabolic parameters, including fasting glucose and insulin, and serum hormone measurements were performed in the central laboratory of the University Medical Centre Groningen (UMCG, Groningen, The Netherlands) after the trial had been finished. Homeostasis model assessment of insulin resistance (HOMA-IR) was calculated as fasting insulin concentration (µU/ml) multiplied by fasting glucose concentration (mmol/l) divided by 22.5 (Matthews *et al.*, 1985). Detailed information, including measurement method, as well as intra- and inter-assay variations of these outcomes, can be found in our previous study investigating effect of a lifestyle intervention on cardiometabolic health in women with infertility and obesity (van Dammen *et al.*, 2018). The steroid hormones A4, T, dihydrotestosterone (DHT), dehydroepiandrosterone (DHEA) and dehydroepiandrosterone sulfate (DHEA-S) were measured using liquid chromatography tandem mass spectrometry (LC-MS/MS) (van der Veen *et al.*, 2019). SHBG was measured with the Architect manufactured by Abbott Diagnostics (Abbott park, IL, USA), using a chemiluminescent micro particle immunoassay. The free androgen index (FAI) was calculated by dividing the total serum T level by the SHBG level and multiplying this by 100. 11β-Hydroxyandrostenedione (11OHA4) and 11KT were analyzed using liquid–liquid extraction followed by LC-MS/MS (Hawley *et al.*, 2020) in Wytheshawe hospital (Manchester, UK**).** AMH was measured with a chemiluminescent microparticle immunoassay on the Roche Cobas 6000 system (Roche, Manheim, Germany). Imprecision for AMH was 2.0%, 2.6%, and 4.9% at 1.66, 4.87, and 8.4 ng/ml, respectively, while the lower limit of quantification was 0.03 ng/ml.

### Statistical analysis

No sample size calculation could be performed owing to the *post hoc* nature of this analysis. Our analyses should therefore be regarded as explorative. Baseline characteristics were compared between RO+ and RO− groups. Data were expressed as mean ± SD or median (interquartile range: IQR) for continuous variables, and proportions (percentage, %) for categorical variables. Normality testing was performed using histograms and normal probability plots (Q-Q plots) combined with the Kolmogorov–Smirnov (K-S) test. The differences between the two groups were compared with Student’s *t*-test or Mann–Whitney *U*-test where relevant for continuous variables and the Chi-square test for categorical variables. Anthropometric measurements and endocrine and metabolic parameters at 3, 3 and 6 months for the two groups were described separately.

**Figure 3.**
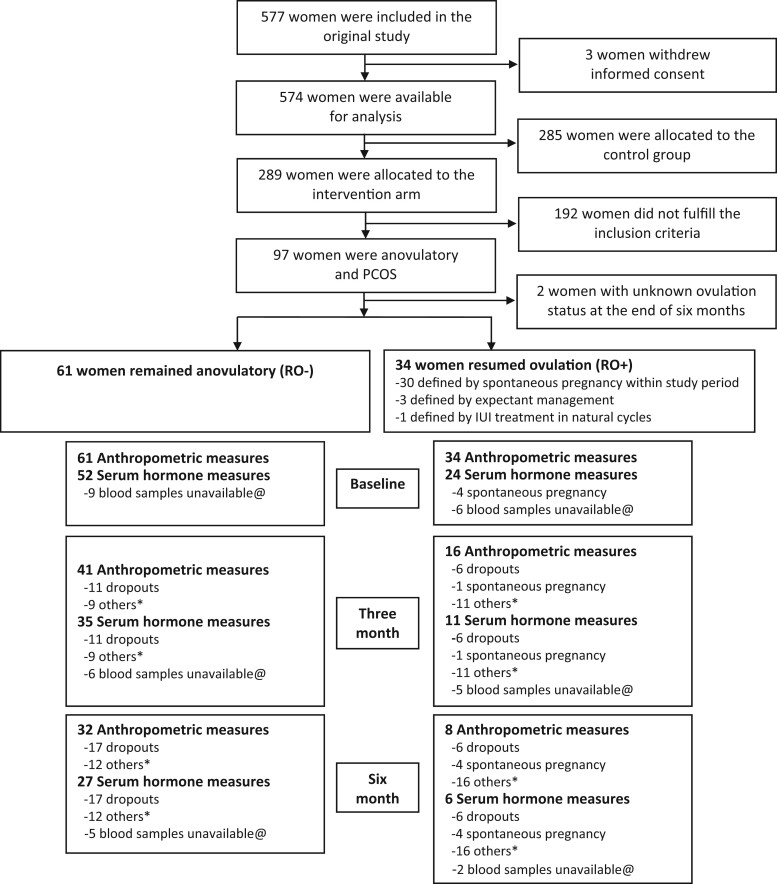
**Flow chart of participants for the current analysis of resumption of ovulation in anovulatory women with PCOS and obesity**. *Failure to attend the hospital visit or other personal reasons. @blood samples were used in other investigations or missing.

It is important to note that while all these women had baseline anthropometric measures (weight, BMI), not all of them had complete data for anthropometric measures or serum hormone measures at 3 or 6 month time points, for various reasons (Fig. 3). We used the generalized estimating equations (GEE) method with exchangeable correlation structure to analyze longitudinal data, which is preferable over complete-case analysis, as the latter does not reflect a real-life situation and poses the risk of selection bias. To answer our research questions, we performed GEE with weight, AMH, HOMA-IR, and androgen concentrations as the dependent variables and with ‘time’ (baseline, 3 months, and 6 months), and group (RO+/RO−) as the independent variables. Subsequently, we added an interaction term between time and group to the models to evaluate differences between the RO+ and RO− groups in the course over time for each of the dependent variables separately. All analyses were corrected for baseline for the respective dependent variable. Moreover, estimated marginal means and SEs by time and group were plotted to visualize patterns of change by group. To identify confounders, we included potential confounders (age, smoking status, education, ethnicity) in the respective GEE models, one at a time. If addition of a confounder changed the effect estimate in the models by more than 10%, the variable was regarded as a relevant confounder. Lastly, we compared the differences in endocrine and metabolic parameters between 3 months and baseline and between 6 months and baseline for the RO+ group and the RO− group separately, to quantify the changes over time within both groups. Only ‘time’ (baseline, 3 months, and 6 months) was included in these models as a categorical independent variable with baseline as the reference. Estimated marginal means at baseline, 3 and 6 months were calculated in the RO+ and RO− groups, respectively.

The Statistical Package for Social Science (IBM SPSS, Armonk, NY, USA, version 27.0) was used to perform statistical analyses and GraphPad Prism (San Diego, CA, USA, version 8.0) was used for data visualization. A *P*-value <0.05 was regarded as statistically significant.

## Results

In total, 577 women were included in the original RCT and 289 women were allocated to the lifestyle intervention arm. Ninety-seven anovulatory women with PCOS who were allocated to the lifestyle intervention arm were selected for the current analysis. After the 6-month lifestyle intervention, two women were excluded owing to unknown ovulation status (lost to follow-up), leaving 61 women in RO+ group and 34 women in RO− group. The flow chart of the current study is shown in Fig. 3.

Table 1 shows baseline measurements of the women included in the current study according to whether or not they resumed ovulation at 6 months. The mean age was 27.5 ± 3.6 years in the RO+ group and 27.9 ± 4.1 years in the RO− group (*P* = 0.65). The mean weight was 101.2 ± 9.5 and 105.0 ± 14.6 kg, respectively (*P* = 0.13). There were no statistically significant differences in other anthropometric measurements and baseline characteristics including ethnicity, education, and smoking status. As for the measured serum hormones, only AMH concentrations at baseline showed statistically significant differences between the RO+ women and the RO− women (median and IQR 4.7 [3.2; 8.3] ng/ml in the RO+ group and 7.2 [5.3; 10.8] ng/ml in the RO− group, *P* = 0.03). Other serum hormone measures, including insulin, HOMA-IR, T, and FAI, did not differ significantly between the two groups. Moreover, none of the variables turned out to be relevant confounders, so we did not further adjust for confounders in the GEE model. Supplementary Table S1 shows endocrine and metabolic parameters at 3 and 6 months in the two groups with decreasing numbers of observations in both groups owing to loss to follow-up over time.

**Table 1.** Baseline characteristics of anovulatory women with PCOS and obesity with *post hoc* allocation into RO+ and RO− groups at the end of 6 months.

	RO+ (n = 34)	RO− (n = 61)	*P*-value
**Anthropometric measurements**			
Weight (kg)	101.2 ± 9.5	105.0 ± 14.6	0.13
BMI (kg/m^2^)	35.5 ± 2.9	36.0 ± 3.6	0.44
Waist circumference (cm)	108.7 ± 7.8	108.0 ± 10.1	0.75
Hip circumference (cm)	122.1 ± 8.2	124.2 ± 8.6	0.26
Waist-hip circumference ratio	0.89 ± 0.06	0.87 ± 0.08	0.28
**Baseline characteristics**			
Age (years)	27.5 ± 3.6	27.9 ± 4.1	0.65
Western European ethnicity	32 (94.1%)	55 (90.2%)	0.51
Current smoker	8 (24.2%)	20 (33.3%)	0.36
Education (number)			0.53
Primary school	1 (2.9%)	3 (4.9%)	
Secondary education	7 (20.6%)	14 (23.0%)	
Intermediate vocational education	20 (58.8%)	26 (42.6%)	
Advanced vocational education or university	5 (14.7%)	12 (19.7%)	
Unknown	1 (2.9%)	6 (9.8%)	
Primary infertility	24 (70.6%)	45 (73.8%)	0.74
**Serum measurements**			
Insulin (pmol/l)	90.2 (70.1; 131.9)	95.5 (73.8; 142.5)	0.48
HOMA-IR	3.1 (2.3; 4.3)	3.3 (2.5; 4.9)	0.54
AMH (ng/ml)	4.7 (3.2; 8.3)	7.2 (5.3; 10.8)	0.03
SHBG (nmol/l)	28.7 (21.3; 46.3)	27.2 (18.3; 38.6)	0.46
LH (U/l)	10.6 ± 4.9	11.8 ± 5.1	0.37
FSH (U/l)	5.0 ± 2.0	5.3 ± 1.6	0.48
A4 (nmol/l)	6.8 ± 2.6	6.8 ± 2.2	0.99
T (nmol/l)	1.6 ± 0.7	1.8 ± 0.8	0.34
DHT (nmol/l)	0.4 ± 0.3	0.3 ± 0.2	0.10
DHEA (nmol/l)	23.3 ± 10.7	19.6 ± 11.0	0.17
DHEA-S (nmol/l)	6.0 ± 2.8	5.2 ± 2.4	0.24
11KT (nmol/l)	1.3 ± 0.5	1.3 ± 0.6	0.69
11OHA4 (nmol/l)	5.1 ± 2.5	4.6 ± 2.4	0.51
FAI	5.5 ± 2.5	7.2 ± 4.1	0.07

RO+: resumed ovulation at the end of 6 months; RO−: remained anovulatory at the end of 6 months; HOMA-IR: homeostatic model assessment for insulin resistance; AMH: anti-Müllerian hormone; SHBG: sex hormone-binding globulin; A4: androstenedione; T: testosterone; DHT: dihydrotestosterone; DHEA: dehydroepiandrosterone; DHEA-S: dehydroepiandrosterone sulfate; 11KT: 11-ketotestosterone; 11OHA4: 11β-hydroxyandrostenedione; FAI: free androgen index.

Data are presented as mean ± SD or median with interquartile (Q25; Q75) or proportion (percentage). The differences between two groups were compared using Fisher’s exact test or chi-square test for categorical variables, and Student’s *t*-test or Mann–Whitney *U* test for continuous variables.

**Figure 4.**
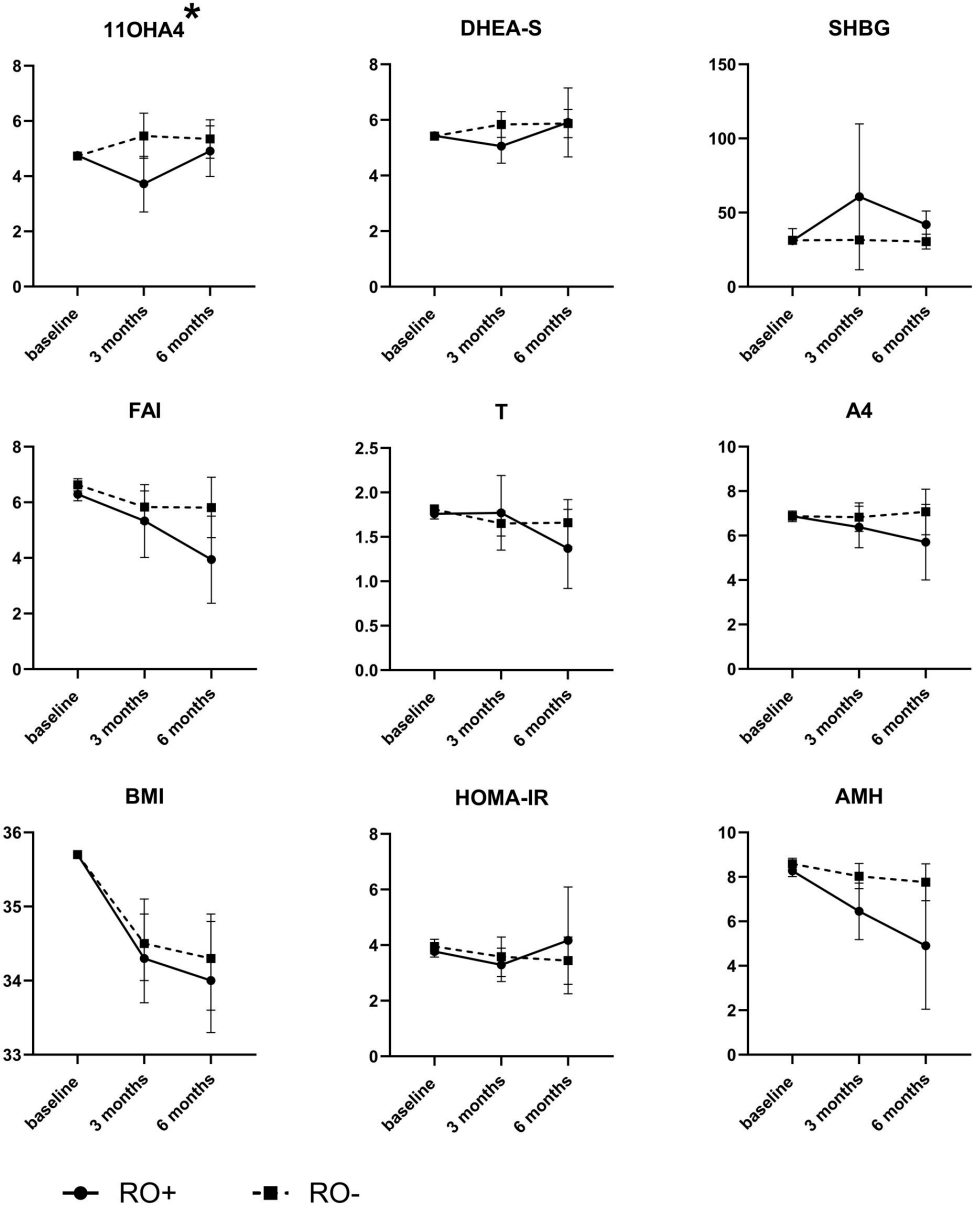
**Baseline-corrected generalized estimating equations analysis of parameters in women who had or had not resumed ovulation after 3 and 6 months of lifestyle intervention**. Data are from women who had (RO+) or had not (RO−) resumed ovulation. GEE: generalized estimating equations, AMH: anti-Müllerian hormone; HOMA-IR: homeostatic model assessment for insulin resistance; SHBG: sex hormone-binding globulin; T: testosterone; A4: androstenedione; FAI: free androgen index; 11OHA4: 11β-hydroxyandrostenedione. *Y*-axes show GEE estimates. Error bars indicate standard errors of GEE estimates. As GEE was performed with correction for baseline, the values at baseline differ from those shown in Table 1. *P < 0.05 for interaction term group and time.

Results of interaction terms between time and group from GEE analyses of weight, AMH, HOMA-IR, and serum hormones at baseline, 3 months and 6 months in two groups are shown in Table 2. The only parameter showing a statistically significant difference over time between the RO+ and RO− groups was 11OHA4 (*P*-value for interaction term between time and RO group: 0.03). A similar trend towards differences over time between the RO+ and RO− groups was observed for SHBG (*P*-value for interaction: 0.07), and DHEA-S (*P*-value for interaction: 0.06). The most pronounced differences were observed in the first 3 months. We did not observe differences between the RO+ and RO− groups for other parameters such as BMI (*P* = 0.85), HOMA-IR (*P* = 0.58) or AMH (*P* = 0.24). For some parameters estimated marginal means and SEs by time and group (Table 2) were also plotted (Fig. 4) to visualize the patterns of change by group.

**Table 2.** Estimated marginal means for anthropometric measurements and endocrine and metabolic parameters from generalized estimating equations analyses.

	Baseline		3 months		6 months		Interaction between time and RO group
	RO+ (n = 34)	RO− (n = 61)	RO+ (n = 16)	RO− (n = 41)	RO+ (n = 8)	RO− (n = 32)	Overall *P*-value
Weight (kg)	103.4 (0.05)	103.4 (0.04)	99.2 (1.1)	100.3 (0.69)	98.5 (1.0)	99.1 (0.87)	0.72
BMI (kg/m^2^)	35.7 (0.01)	35.7 (0.01)	34.3 (0.3)	34.5 (0.3)	34.0 (0.4)	34.3 (0.3)	0.85
Waist circumference (cm)	108.2 (0.16)	108.1 (0.15)	103.2 (2.2)	105.5 (0.8)	102.8 (2.2)	103.5 (1.1)	0.60
Hip circumference (cm)	123.3 (0.04)	123.3 (0.03)	121.3 (1.7)	121.4 (0.8)	120.0 (1.8)	119.3 (0.9)	0.89
Waist-hip circumference ratio	0.88 (0.002)	0.88 (0.001)	0.85 (0.01)	0.87 (0.01)	0.87 (0.01)	0.87 (0.01)	0.29
Insulin (pmol/l)	109.9 (2.6)	113.8 (2.9)	95.7 (9.7)	101.8 (9.0)	117.4 (24.0)	100.0 (11.0)	0.57
HOMA-IR	3.8 (0.1)	3.9 (0.1)	3.3 (0.3)	3.6 (0.4)	4.2 (1.0)	3.4 (0.4)	0.58
AMH (ng/ml)	8.3 (0.1)	8.6 (0.1)	6.4 (0.6)	8.0 (0.3)	4.9 (1.5)	7.8 (0.4)	0.24
SHBG (nmol/l)	31.1 (0.5)	31.3 (0.2)	60.6 (25)	31.6 (1.2)	41.9 (4.6)	30.4 (2.5)	0.07
LH (U/l)	11.3 (0.3)	11.6 (0.2)	9.1 (1.5)	10.6 (0.8)	8.9 (2.9)	11.2 (1.0)	0.67
FSH (U/l)	5.1 (0.1)	5.1 (0.06)	4.0 (0.6)	4.8 (0.3)	3.7 (1.0)	5.1 (0.3)	0.27
A4 (nmol/l)	6.9 (0.1)	6.9 (0.1)	6.4 (0.5)	6.8 (0.3)	5.7 (0.9)	7.1 (0.5)	0.36
T (nmol/l)	1.8 (0.03)	1.8 (0.03)	1.7 (0.2)	1.6 (0.07)	1.4 (0.2)	1.7 (0.1)	0.50
DHT (nmol/l)	0.35 (0.01)	0.33 (0.004)	0.34 (0.06)	0.31 (0.02)	0.27 (0.07)	0.37 (0.03)	0.33
DHEA (nmol/l)	21.1 (0.3)	20.6 (0.2)	18.9 (3.1)	25.3 (2.3)	26.9 (6.2)	20.7 (2.0)	0.13
DHEA-S (nmol/l)	5.4 (0.02)	5.4 (0.01)	5.1 (0.3)	5.8 (0.2)	5.9 (0.6)	5.9 (0.3)	0.06
11KT (nmol/l)	1.3 (0.02)	1.3 (0.02)	1.1 (0.2)	1.3 (0.1)	1.3 (0.2)	1.7 (0.4)	0.65
11OHA4 (nmol/l)	4.7 (0.03)	4.7 (0.01)	3.7 (0.5)	5.5 (0.4)	4.9 (0.5)	5.3 (0.4)	0.03
FAI	6.3 (0.1)	6.6 (0.1)	5.3 (0.7)	5.8 (0.3)	3.9(0.8)	5.8 (0.6)	0.33

GEE: generalized estimating equations; HOMA-IR: homeostatic model assessment for insulin resistance; AMH: anti-Müllerian hormone; SHBG: sex hormone-binding globulin; A4: androstenedione; T: testosterone; DHT: dihydrotestosterone; DHEA: dehydroepiandrosterone; DHEA-S: dehydroepiandrosterone sulfate; 11KT: 11-ketotestosterone; 11OHA4: 11β-hydroxyandrostenedione; FAI: free androgen index.

Data are presented as estimated marginal means (SE) from GEE analyses. The analyses are corrected for baseline, with time and group as the independent variables and an interaction term for time × group.

In Table 3 we show the estimated marginal means of weight, AMH, HOMA-IR, and serum hormone concentrations at 3 and 6 months for RO+ and RO− groups separately, to quantify the changes over time within both groups. Weight and BMI significantly decreased during the course of the lifestyle intervention for both groups (*P* < 0.001). AMH concentrations were lower at 3 and 6 months compared to baseline in the RO+ group (baseline estimated marginal means and SE 6.7 ng/ml [1.0] versus 3 months: 4.7 [0.9], *P* = 0.01, and 6.7 [1.0] versus 6 months: 3.0 [1.2], *P* = 0.02) but not in the RO− group (baseline: 8.8 [0.9], 3 months: 8.3 [0.8], and 6 months: 7.9 [0.8]). FAI was significantly lower at 6 months compared to baseline in the RO+ group (baseline 5.5 [0.5] versus 6 months: 3.5 [0.5], *P* = 0.003) and in the RO− group (baseline: 7.2 [0.6], 3 months: 6.0 [0.4], and 6 months: 5.9 [0.5]; *P* = 0.007 for the difference between 3 months and baseline and *P* = 0.049 for the difference between 6 months and baseline). There were no statistically significant differences between 3 months and baseline or between 6 months and baseline for either SHBG or DHEA-S concentrations for both RO+ and RO− groups.

**Table 3.** Estimated marginal means for anthropometric measurements and endocrine and metabolic parameters at baseline, 3 months, and 6 months after starting lifestyle intervention, stratified by group.

	RO+					RO−				
	Baseline	3 months	*P*-value	6 months	*P*-value	Baseline	3 months	*P*-value	6 months	*P*-value
Weight (kg)	101.2 (1.6)	97.0 (1.9)	<0.001	96.1 (1.9)	<0.001	105.0 (1.9)	101.7 (1.9)	<0.001	100.4	<0.001
BMI (kg/m^2^)	35.5 (0.5)	34.1 (0.6)	<0.001	33.7 (0.6)	<0.001	36.0 (0.5)	34.8 (0.5)	<0.001	34.5 (0.5)	<0.001
Waist circumference (cm)	108.7 (1.3)	104.0 (2.0)	0.03	104.1 (1.9)	0.03	108.2 (1.3)	105.4 (1.3)	0.001	103.8 (1.4)	<0.001
Hip circumference (cm)	122.1 (1.4)	119.8 (2.1)	0.18	118.6 (2.0)	0.04	124.3 (1.1)	122.3 (1.3)	0.02	120.2 (1.2)	<0.001
Waist-hip circumference ratio	0.89 (0.01)	0.86 (0.02)	0.04	0.89 (0.01)	0.77	0.87 (0.01)	0.86 (0.01)	0.27	0.86 (0.01)	0.44
Insulin (pmol/l)	103.1 (8.5)	85.2 (11.8)	0.09	106.3 (22.8)	0.90	116.7 (9.5)	103.0 (10.8)	0.16	103.1 (9.3)	0.23
HOMA-IR	3.5 (0.3)	2.8 (0.4)	0.045	3.7 (0.9)	0.85	4.0 (0.4)	3.6 (0.4)	0.26	3.5 (0.3)	0.24
AMH (ng/ml)	6.7 (1.0)	4.7 (0.9)	0.01	3.0 (1.2)	0.02	8.8 (0.9)	8.3 (0.8)	0.12	7.9 (0.8)	0.08
SHBG (nmol/l)	33.4 (3.9)	62.2 (27.2)	0.28	45.7 (9.2)	0.14	30.5 (2.4)	30.5 (2.1)	0.98	31.0 (2.4)	0.82
LH (U/l)	10.6 (1.0)	9.1 (1.8)	0.37	8.9 (2.5)	0.53	11.7 (0.7)	10.7 (0.8)	0.20	11.1 (0.9)	0.55
FSH (U/l)	5.0 (0.4)	3.7 (0.7)	0.08	3.7 (0.8)	0.19	5.3 (0.2)	4.9 (0.3)	0.19	5.1 (0.3)	0.53
A4 (nmol/l)	6.8 (0.5)	6.4 (0.4)	0.43	5.8 (0.8)	0.27	6.8 (0.3)	6.7 (0.4)	0.84	7.0 (0.5)	0.63
T (nmol/l)	1.6 (0.1)	1.6 (0.2)	0.99	1.3 (0.2)	0.26	1.8 (0.1)	1.6 (0.1)	0.01	1.7 (0.1)	0.26
DHT (nmol/l)	0.4 (0.05)	0.4 (0.06)	0.73	0.3 (0.05)	0.08	0.3 (0.02)	0.3 (0.03)	0.33	0.4 (0.03)	0.17
DHEA (nmol/l)	23.3 (2.1)	20.9 (3.2)	0.48	27.5 (7.0)	0.48	20.0 (1.6)	25.1 (2.8)	0.04	20.6 (2.2)	0.75
DHEA-S (nmol/l)	6.0 (0.6)	5.5 (0.6)	0.17	6.3 (0.8)	0.71	5.2 (0.3)	5.7 (0.4)	0.06	5.7 (0.4)	0.08
11KT (nmol/l)	1.3 (0.1)	1.1 (0.2)	0.26	1.3 (0.2)	0.93	1.3 (0.08)	1.3 (0.1)	0.90	1.6 (0.4)	0.48
11OHA4 (nmol/ll)	5.1 (0.5)	4.3 (0.6)	0.14	5.3 (0.8)	0.74	4.7 (0.3)	5.6 (0.5)	0.05	5.2 (0.4)	0.24
FAI	5.5 (0.5)	4.8 (0.7)	0.3	3.5 (0.5)	0.003	7.2 (0.6)	6.0 (0.4)	0.007	5.9 (0.5)	0.049

RO+: resumed ovulation at the end of 6 months; RO: remained anovulatory at the end of 6 months; HOMA-IR: homeostatic model assessment for insulin resistance; AMH: anti-Müllerian hormone; SHBG: sex hormone-binding globulin; A4: androstenedione; T: testosterone; DHT: dihydrotestosterone; DHEA: dehydroepiandrosterone; DHEA-S: dehydroepiandrosterone sulfate; 11KT: 11-ketotestosterone; 11OHA4: 11β-hydroxyandrostenedione; FAI: free androgen index.

Data are presented as estimated marginal means (SE) from generalized estimating equations analyses. *P*-values refer to change from baseline at 3 months and at 6 months in RO+ and RO− women, respectively.

## Discussion

In a *post hoc* analysis of our LIFEstyle RCT, we investigated if resumption of ovulation after a 6-month lifestyle intervention in women with PCOS and obesity was associated with changes in endocrine and metabolic parameters (weight, insulin resistance, AMH, and androgens). At baseline, RO+ women had lower serum AMH concentrations than RO− women. During and after lifestyle intervention, only 11OHA4 showed a statistically significant difference between the RO+ and RO− groups over the course of the intervention, while SHBG and DHEA-S showed a similar trend. Moreover, RO+ women showed a significant reduction of serum AMH concentrations at 3 and 6 months compared to baseline measures while RO− women did not.

The association between greater degree of weight loss and restored ovarian function in anovulatory women with PCOS has been supported by evidence from a small study involving bariatric surgery in 12 anovulatory women with PCOS, in which all women regained normal ovulation and menstrual cycles after a weight loss of 41 kg on average (Escobar-Morreale *et al.*, 2005). Previous studies of lifestyle intervention in women with PCOS and obesity demonstrated that, compared to non-responders, those women who resumed ovulation after weight loss had experienced greater reductions in weight/BMI (Kuchenbecker *et al.*, 2011; Jarrett and Lujan, 2016). Although the optimal degree of weight loss to restore ovulatory function is not clear, at least 5–10% weight loss is generally recommended (Norman *et al.*, 2004; Teede *et al.*, 2018). In the current study, there was no significant difference between the RO+ and RO− groups over the course of the intervention for body weight or BMI. This might be explained by the small sample size because of attrition of participants in our study. Additionally, both insulin concentrations and insulin resistance represented by HOMA-IR did not show significant differences in RO+ and RO− women. Possible explanations might include the limited statistical power of the current study, the high average BMI within our population (BMI > 35 kg/m^2^), or the limited degree of weight reduction. It is important to note that moderate weight loss in women with a high BMI does not consistently lead to an improvement in insulin resistance (Tang *et al.*, 2006).

Hyperandrogenism is one of the most dominant features of PCOS (Azziz *et al.*, 2006). However, biochemical hyperandrogenism remains a diagnostic challenge and recent research advocates the quantification of several steroid hormones simultaneously using LC-MS/MS (Keevil, 2014, 2019). For instance, it has been demonstrated that 11-oxygenated androgens represent the majority of circulating androgens in women with PCOS. Consequently, focus has recently shifted towards including these 11-oxygenated C19 steroids in biochemical hyperandrogenism analysis, although definitive conclusions have not been reached (O'Reilly *et al.*, 2017; Hawley *et al.*, 2020). Therefore, there is room for discussion as to which androgen or combination of androgens is a better marker associated with restored ovulation in anovulatory women with PCOS and obesity after weight loss. Weight loss results in decreased androgen production in the ovarian theca cells owing to a reduction in insulin as co-factor to LH. The combination of reduced insulin and androgens concentrations consequently leads to more SHBG production in the liver (Poretsky, 1991). In our study, 11OHA4 was the only parameter that showed a statistically significant difference between the RO+ and RO− groups over the course of the intervention. Regarding 11OHA4, as one of the most abundant unconjugated androgens produced by the human adrenals, our results imply a contribution of adrenal hormones in this group of women with obesity and PCOS. We also found a trend towards significant differences in SHBG and DHEA-S between two groups. This is somewhat in line with a previous study in women with PCOS undergoing a weight loss program (Guzick *et al.*, 1994). Moreover, FAI in both groups decreased over the course of the intervention period although no significant differences between groups were found. All these results suggest that there is an important role of androgen concentrations, especially 11OHA4, in restoring ovulation in women with PCOS but this needs to be explored in larger studies in the future.

Furthermore, we did not observe a significant interaction between time and group for serum AMH. Serum AMH is considered a stable marker of the oocyte pool with limited variation throughout the menstrual cycle (Broer *et al.*, 2014). Data regarding the association between AMH changes and resumption of ovulation after weight loss intervention in women with PCOS and obesity are limited. Thomson *et al.* (2009) has demonstrated that in a 20-week weight loss program (a hypocaloric dietary with 1430 kcal per day) in 52 women, changes in AMH concentrations did not significantly differ for women who responded with improved reproductive function (identified as having either improvement in ovulatory function and/or improvements in menstrual status) and those who did not. There are some differences between our studies: First, inconsistencies exist in the measurement of ovulation across studies. In our study, we solely used resumption of ovulation as the indicator, whereas in their study, either improvement in ovulatory function and/or improvements in menstrual status were considered. Second, is heterogeneity in lifestyle interventions. Indeed, there is evidence from one study suggesting that the response of serum AMH concentrations to interventions, such as diet, exercise, and a combination of diet and exercise, may vary in women with PCOS and obesity. Specifically, serum AMH concentrations were significantly decreased only in the diet group, but not in the exercise group or the combined intervention group (Nybacka *et al.*, 2013). Third, there is heterogeneity in the diagnosis of PCOS. Although both studies used the Rotterdam criteria to diagnose PCOS and baseline body weight and age were similar, potential differences in other characteristics cannot be ruled out as PCOS is such a multi-phenotype syndrome. Fourth, there were differences in methods of data analysis. Thomson *et al.* (2009) calculated the changes of AMH concentrations before and after the intervention and compared changes in concentrations directly between two groups, while we compared the concentrations during and after the intervention with the correction for baseline measurements. Notably, we revealed a significant reduction in serum AMH concentrations among RO+ women at 3 and 6 months compared to baseline measures while this was not observed in RO− women. It is reasonable to assume that no significant differences in AMH concentrations over time were detected between the two groups, possibly because of the small sample size. We clearly need more studies to investigate the changes in AMH concentrations and their putative association with the resumption of ovulatory cycles following weight loss in a range of PCOS phenotypes (Azziz *et al.*, 2006). However, we can get a glimpse of possible results by looking at the existing research on bariatric surgery, which yields greater weight loss than lifestyle intervention. Bariatric surgery has been shown to reduce AMH concentrations in 14 women with PCOS and is associated with weight loss (Chiofalo *et al.*, 2017). Importantly, both the Thomson *et al.* (2009) study and our study showed lower AMH concentrations at baseline in women with improved ovulation status than non-responders. This might suggest, hypothetically, that anovulatory women with higher baseline AMH concentrations would require greater weight loss in order to decrease AMH concentrations and facilitate the restoration of ovulation.

The mechanism of resumption of ovulation in women with obesity and PCOS after weight loss is far from clear. Our results suggest an important role for reduction of androgens concentrations, especially 11-oxygenated androgens, in the resumption of ovulation. With the conduct of larger-scale studies, this can presumably provide significant clinical guidance for anovulatory women with PCOS, i.e., monitoring androgens concentrations to provide individualized recommendations for lifestyle interventions (amount of weight loss) before ovulatory cycles resume.

The strength of this study is the use of well-characterized participants of an RCT, close follow-up of participants during and after the lifestyle program, and the measurement of a wide range of steroid hormones, including adrenal oxygenated steroids using LC–MS/MS methods. However, several limitations in this study need to be mentioned. First, no direct observation of follicular growth and rupture by high-resolution transvaginal ultrasonography was performed to confirm ovulations for all women with PCOS at the end of lifestyle program. We did not evaluate ovulation status in the third month after randomization thus we were unable to investigate the association over time between resumption of ovulation and weight loss or insulin resistance improvement at 3 months. Second, the number of available serum hormone measurements decreased over the course of the intervention period owing to the nature of the RCT with dropouts during the intervention, pregnancies, missed hospital visits, or blood samples being exhausted for other measurements. The remaining relatively small sample size might produce a false negative result. Third, we could not perform subgroup analyses to explore the optimal degree of weight loss required to restore ovulatory function in PCOS owing to the small sample size. The nature of the study design also did not allow us to explore the causal relation between lower androgen concentrations and ovulation resumption. Last, we cannot divide the subtypes of PCOS (Azziz *et al.*, 2006) in this analysis and our results cannot be generalized to other populations of anovulatory PCOS women without obesity.

In conclusion, in this *post hoc* exploratory analysis, we showed that 11OHA4 was the only parameter showing a statistically significant difference between the RO+ and RO− groups over the course of the intervention, although a similar trend was observed for DHEA-S and SHBG. We have also observed that RO+ women had lower concentrations of AMH than RO− women at baseline. A significant reduction in serum AMH concentrations was shown among RO+ women at 3 and 6 months compared to baseline measures while this was not observed in RO− women. In the future, if our results can be confirmed in other studies, monitoring of androgen concentrations during lifestyle intervention may help to provide individualized recommendations on resumption of ovulatory cycles in anovulatory women with PCOS and obesity.

## Supplementary Material

deae058_Supplementary_Data

## Data Availability

The data underlying this article will be shared on reasonable request to the corresponding author.
