# Supplementary material for: Resumption of ovulation in anovulatory women with PCOS and obesity is associated with reduction of 11β-hydroxyandrostenedione concentrations
Source: Hum Reprod. 2024 Mar 19;39(5):1078–88. doi: 10.1093/humrep/deae058 (PMC11063562; doi:10.1093/humrep/deae058)
Supplement: deae058_Supplementary_Data [file deae058_supplementary_data.pdf]

OXFORD
human reproduction

Human Reproduction, 2024, 1–1
https://doi.org/10.1093/humrep/deae058
Supplementary Data

Supplementary Table S1. Anthropometric measurements and endocrine and metabolic parameters at 3 and 6 months in anovulatory women with PCOS and obesity with *post hoc* allocation into RO+ and RO− at the end of 6 months, raw data.

	Three months		Six months	
	**RO+ (n = 16)**	**RO− (n = 41)**	**RO+ (n = 8)**	**RO− (n = 32)**
**Anthropometric measurements**				
Weight (kg)	97.5 ± 9.3	101.0 ± 13.8	98.5 ± 9.8	98.8 ± 12.5
BMI (kg/m^2)	34.4 ± 3.0	34.4 ± 4.0	34.0 ± 3.2	34.2 ± 3.6
Waist circumference (cm)	104.1 ± 8.2	104.9 ± 9.6	105.1 ± 6.3	103.6 ± 8.7
Hip circumference (cm)	119.2 ± 9.4	121.9 ± 9.3	120.7 ± 8.1	120.0 ± 7.6
Waist-hip circumference ratio	0.87 ± 0.07	0.86 ± 0.07	0.89 ± 0.04	0.86 ± 0.07
**Serum measurements**				
Insulin (pmol/l)	61.1 (54.0; 113.2)	75.0 (56.2; 122.2)	83.0 (53.3; 128.0)	82.3 (58.0; 147.0)
HOMA-IR	1.8 (1.8; 3.7)	2.8 (1.8; 4.3)	2.7 (1.8; 4.3)	2.8 (1.8; 4.9)
AMH (ng/ml)	3.9 (3.5; 7.8)	6.4 (4.8; 10.3)	5.3 (3.0; 10.3)	6.4 (5.3; 10.3)
SHBG (nmol/l)	30.0 (26.5; 54.5)	30.6 (22.8; 36.6)	31.6 (27.0; 50.1)	31.5 (22.7; 37.7)
LH (U/l)	9.3 ± 6.4	10.5 ± 5.2	9.3 ± 6.4	11.1 ± 4.6
FSH (U/l)	3.7 ± 2.4	4.8 ± 1.6	3.8 ± 2.2	5.1 ± 1.7
A4 (nmol/l)	6.8 ± 1.8	6.7 ± 2.3	5.8 ± 2.5	7.4 ± 2.9
T (nmol/l)	1.8 ± 0.9	1.6 ± 0.7	1.4 ± 0.7	1.8 ± 0.7
DHT (nmol/l)	0.4 ± 0.2	0.3 ± 0.2	0.3 ± 0.1	0.4 ± 0.2
DHEA (nmol/l)	22.3 ± 13.0	25.1 ± 17.0	28.3 ± 26.5	22.1 ± 12.8
DHEA-S (nmol/l)	5.1 ± 2.8	5.7 ± 2.5	5.9 ± 2.8	5.7 ± 2.5
11KT (nmol/l)	1.3 ± 0.5	1.3 ± 0.6	1.4 ± 0.7	1.6 ± 1.8
11OHA4 (nmol/l)	4.9 ± 2.0	5.5 ± 2.9	5.6 ± 2.8	4.9 ± 2.2
FAI	4.9 ± 2.6	5.8 ± 2.5	3.7 ± 1.2	5.8 ± 2.4

Data are presented as mean § SD or median with interquartile (Q25; Q75) or proportion (percent).
RO+: resumed ovulation at the end of 6 months; RO−: remained anovulatory at the end of 6 months; HOMA-IR: homeostatic model assessment for insulin resistance; AMH: anti-Müllerian hormone; SHBG: sex hormone-binding globulin; A4: androstenedione; T: testosterone; DHT: dihydrotestosterone; DHEA: dehydroepiandrosterone; DHEA-S: dehydroepiandrosterone sulfate; 11KT: 11-ketotestosterone; 11OHA4: 11β-hydroxyandrostenedione; FAI: free androgen index.
